# Unveiling the inflammatory potential of endogenous sncRNAs: Insights from infections to autoimmune diseases

**DOI:** 10.1016/j.omtn.2024.102297

**Published:** 2024-08-23

**Authors:** Taisuke Kanaji, Xiang-Lei Yang

**Affiliations:** 1 Department of Molecular Medicine, The Scripps Research Institute, La Jolla, CA 92037, USA

## Main text

**Figure 1:**
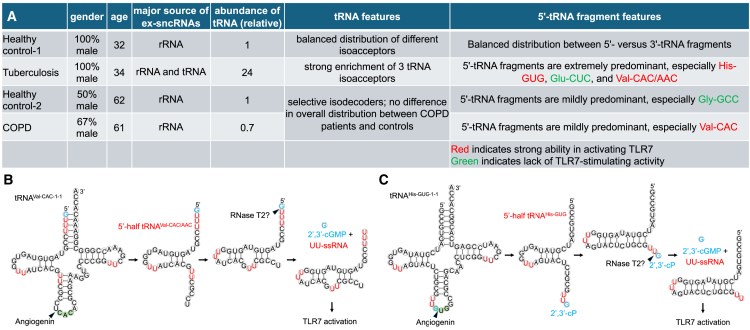
5'-tRNA fragments enriched in patients' plasma and the proposed mechanims for their TLR7 stimulating activity (A) Summary of circulating sncRNAs detected in the plasma of patients with tuberculosis infection and chronic obstructive pulmonary disease (COPD). (B and C) The proposed mechanism for the activation of tRNA-Val-CAC/AAC (B) and tRNA-His-GUG (C) for endosomal TLR7 stimulation. Two RNase cleavage steps produce a guanosine 2′,3′-cyclic phosphate (2′,3′-cGMP) and a consecutive uridine-containing single-stranded RNA (UU-ssRNA) from each tRNA to occupy the two binding sites of TLR7.

In two separate papers from the Kirino laboratory, Gumas et al. and Shigematsu et al., respectively, reported comprehensive investigations of circulating short non-coding RNAs (sncRNAs) presented in the plasma of patients with *Mycobacterium tuberculosis* (Mtb) infection and chronic obstructive pulmonary disease (COPD).^1^^,^^2^ Major circulating species of sncRNAs include microRNAs (miRNAs), small nuclear RNAs (snRNAs), small nucleolar RNAs (snoRNAs), and fragments derived from tRNA and rRNA. However, previous transcriptome studies of circulating sncRNAs did not capture most of the tRNA and rRNA fragments because they have ends non-compatible with the standard RNA sequencing (RNA-seq) technology, such as 2′,3′-cyclic phosphate (cP). By pre-treating plasma RNA with T4 polynucleotide kinase to convert all RNA ends to 5′-monophosphate (P) and 3′-hydroxyl (OH), the authors captured the entire circulating sncRNA transcriptome in human plasma samples for the first time. Despite the limited clinical samples, they found that circulating sncRNAs overall are significantly increased in Mtb-infected patients, particularly those derived from three tRNA isoacceptors, including 5′-tRNA His-GUG and 5′-tRNA Val-CAC/AAC, but not 3′-tRNA fragments (Figure 1A).^1^ In contrast, patients with COPD showed no significant increase in the total amount of circulating sncRNAs or tRNA, and 5′- versus 3′-tRNA fragments are more or less balanced in their distributions; nevertheless, the 5′-tRNA Val-CAC was also found to be significantly accumulated in the plasma of patients with COPD (Figure 1A).^2^ Interestingly, both 5′-tRNA His-GUG and 5′-tRNA Val-CAC/AAC, but not 3′-tRNA Val-CAC/AAC, can activate macrophages through the single-stranded RNA (ssRNA) receptor Toll-like receptor (TLR)7,^3^^,^^4^ inducing the production of inflammatory cytokines. This suggests that the induction of the 5′-tRNA fragments is linked to their immune-activation capacity. A selective mechanism may exist in producing or stabilizing immune-active molecules under an inflammatory disease state. The selective enrichment of these immune-active tRNA fragments, especially in Mtb-infected patients, might be part of the body’s defense mechanism against the infection.

Unlike *in*-*vitro*-transcribed RNAs, endogenous tRNAs are known to have weaker abilities in activating TLR7 due to their base modifications.^5^ Incorporating specific modifications found in tRNAs into the mRNA vaccines for COVID-19 has helped to mitigate severe immune responses that could be harmful. However, work from the Kirino lab demonstrated that the 5′-tRNA His-GUG half with natural base modifications maintains a similar potency to their corresponding unmodified version in stimulating TLR7^3^; the 5′-tRNA Val-CAC/AAC half with natural modifications can also stimulate TLR7, although the activity is reduced.^4^ The TLR7 stimulation activity of endogenous 5′-fragments and the higher potency of the endogenous 5′-tRNA His-GUG relative to the 5′-tRNA Val-CAC/AAC were further confirmed by using antisense oligonucleotides specifically targeting each fragment.^3^^,^^4^ These studies collectively underscore the intriguing potential of endogenous tRNA fragments to induce inflammation.^1^^,^^2^^,^^3^^,^^4^

Although full-length tRNAs were also reported to be abundantly present in human plasma, they were not considered in the sncRNA analysis. Full-length tRNAs lack TLR7-stimulating activity. TLR7 possesses two binding sites, one for guanosine and the other for a successive uridine-containing ssRNA. Angiogenin (ANG) is an endoribonuclease often involved in cleaving tRNA (and rRNA). ANG-mediated cleavage leaves the 3′ end with a 2′,3′-cP, and guanosine with cP (G-cP) exhibits significantly higher affinity to TLR7 compared to guanosine without cP. A 5′-GUUU motif was found in 5′-tRNA Val-CAC/AAC as a universal sequence signature for activating TLR7^4^ (Figure 1B). In contrast, the 5′-tRNA His-GUG half contains a UUG-cP motif at the 3′ end after ANG cleavage (Figure 1C). The authors suggested that additional cleavage of the 5′-GUUU or UUG-cP-3′ motif inside the endosome to release both a G-cP and a successive uridine-containing ssRNA might explain their TLR7-stimulating activities (Figures 1B and 1C).

These studies significantly impact our understanding of immune response and potential mechanisms underlying autoimmune diseases. Using the monocyte-derived cell line THP-1, the authors demonstrated that tRNA fragments can induce the secretion of inflammatory cytokines such as tumor necrosis factor alpha (TNF-α) and interleukin (IL)-1β via TLR7, thereby enhancing bactericidal effects. Of note, TLR7 expression in monocytes and macrophages is lower than that in plasmacytoid dendritic cells (pDCs), which is a unique subset of dendritic cells implicated in the initiation and development of many autoimmune diseases. Recent findings suggest that excessive TLR7 activation due to mutations can lead to autoimmune diseases like lupus.^6^ If these tRNA fragments activate pDCs with high TLR7 expression, then it could lead to a strong secretion of type I interferons, potentially causing autoimmune tissue damage. Thus, the inflammatory nature of these endogenous tRNA fragments highlights the necessity to explore the potential implications of sncRNAs as the contributor to the pathogenesis of autoimmune diseases.^7^

Future research based on these results is crucial to investigate the dynamics of plasma sncRNAs in various infectious diseases or chronic inflammatory conditions. It will be particularly interesting to explore the impact of viral infections (like COVID-19) compared to bacterial infections (like tuberculosis) on circulating sncRNAs. Additionally, the mechanism behind the selective enrichment of certain sncRNAs under an inflammatory disease state— whether it is due to increased cleavage of full-length tRNA by RNases, increased expression of tRNA itself, or increased secretion of tRNA—remains unclear. Further research is urgently needed to elucidate this mechanism.
